# Myocardium at risk quantified by contrast enhanced steady-state free precession does not differ in extent or severity when comparing patients with ST-elevation myocardial infarction treated with standard reperfusion or postconditioning

**DOI:** 10.1186/1532-429X-17-S1-P105

**Published:** 2015-02-03

**Authors:** Peder Sörensson, Marcus Carlsson, Martin Ugander, Håkan Arheden, John Pernow

**Affiliations:** Department of Molecular Medicine and Surgery, Karolinska Institutet, Stockholm, Sweden; Department of Clinical Physiology, Lund University Hospital, Lund, Sweden; Department of Medicine, Karolinska Institutet, Stockholm, Sweden

## Background

Accurate quantification of the ischemic myocardium at risk (MaR) in patients with ST- elevation myocardial infarction (STEMI) is paramount in studies of therapies seeking to reduce infarct size expressed as percent of MaR. Cardiac magnetic resonance (CMR) is an important clinical research tool for determination of MaR and infarct size in the same examination within one week after STEMI. Recent data suggest that cardioprotective interventions such as ischemic postconditioning or remote conditioning reduce infarct size, but may also reduce the extent and severity of edema detected with T2-weighted imaging. Such an effect might limit the value of determining MaR using T2-weighted sequences. Contrast enhanced steady-state free precession (CE-SSFP) imaging for quantifying MaR in STEMI has previously been validated compared to single photon emission computed tomography following radiotracer injection prior to reperfusion. The aim of the current study was to compare extent and severity of signal intensity changes in MaR determined from CE-SSFP in patients with STEMI randomized to postconditioning or standard percutaneous coronary intervention (PCI).

## Methods

**Figure 1:**
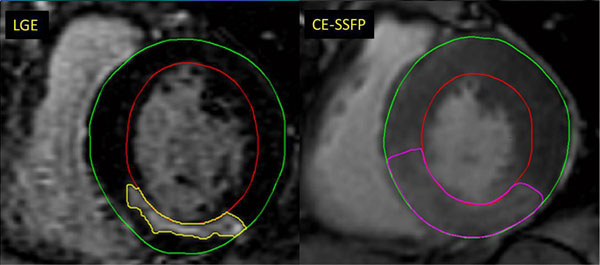
**Corresponding left ventricular short axis views from a patient with inferior myocardial infarction.** Left panel, infarct size images with late gadolinium enhancement (LGE) and right panel, myocardium at risk determined by contrast enhanced steady-state free precession at end-systole (CE-SSFP).

Patients (n=76, age 37-87 years) eligible for primary PCI due to STEMI were randomized to standard PCI (controls, n=38) or postconditioning (n=38) consisting of four cycles of 60 sec reperfusion and 60 sec of re-occlusion before permanent reperfusion. CMR (1.5T Signa Excite Twin Speed, General Electric Healthcare, Waukesha, Wisconsin, USA) was performed 7.8±1.2 days after myocardial infarction using a protocol in which the contrast agent (0.2 mmol/kg; Omniscan, GE Healthcare) was administered before acquisition of short-axis CE-SSFP images. MaR was determined by manual delineation, in end-diastole and end-systole, as the contrast-enhanced myocardial volume in relation to left ventricular mass (Figure 1). A subgroup of patients underwent quantification of the signal intensity ratio of the contrast enhanced MaR region divided by a remote region.

## Results

**Figure 2:**
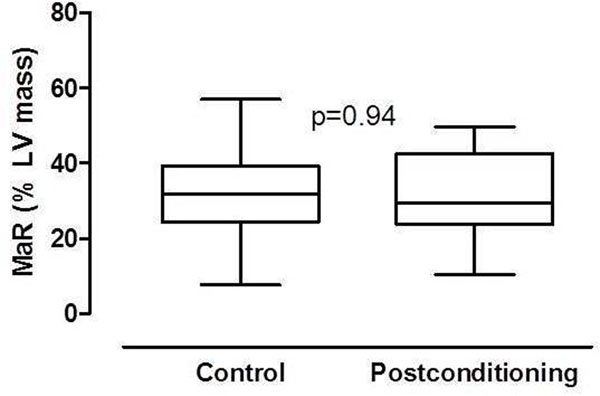
Box-plot with whiskers of myocardium at risk (MaR) expressed as percent of left ventricle (LV) mass. MaR did not differ between patients in the control (n=38) and postconditioning (n=38) groups.

MaR size did not differ between postconditioning and controls (mean±SD, 31.9±10.5% vs 32.1±12.2%, p=0.94) (Figure 2). The MaR signal intensity ratio did not differ between postconditioning (n=20, 1.8±0.5) and controls (n=20, 1.8±0.4, p=0.88). The interobserver variability (n=12) of MaR size by CE-SSFP was 1.4±3.1% of left ventricular mass.

## Conclusions

Postconditioning affects neither the extent nor severity of signal intensity changes in MaR quantified by CE-SSFP performed one week after STEMI. This indicates that CE-SSFP is an accurate and appropriate imaging method to quantify MaR in patients with STEMI, even in the setting of treatment with postconditioning.

## Funding

Swedish Heart and Lung Foundation.

